# Intravaginal Delivery of Oxybutynin: An Alternative Administration Route to Improve its Pharmacokinetic and Pharmacodynamic Effects

**DOI:** 10.1016/j.euros.2026.07.004

**Published:** 2026-07-23

**Authors:** Sophie I. Peltenburg, Anouk C. Meijs, Lisa Pagan, Emily Somohardjo, Henk W. Elzevier, Janneke I.M. van Uhm, Marije E. Otto, Maria J. Juachon, Pamela Strugala, Alexa J. Tibboel, Koos Burggraaf, Bart W.J. Hellebrekers, Naomi B. Klarenbeek

**Affiliations:** a Centre for Human Drug Research, Leiden, The Netherlands; b Clinical Pharmacology Department, Leiden University Medical Centre, Leiden, The Netherlands; c Urology Department, Leiden University Medical Centre, Leiden, The Netherlands; d Leiden Academic Centre for Drug Research, Leiden, The Netherlands; e Gynaecology Department, Haga Hospital, The Hague, The Netherlands

**Keywords:** Anticholinergics, Dry mouth, FemTech, Intravaginal administration, MedRing, Metabolite/parent ratio, OAB, Overactive bladder, Oxybutynin, Side effects

## Abstract

**Background and objective:**

While oxybutynin is the most efficacious oral antimuscarinic treatment to reduce incontinence episodes in patients with an overactive bladder, oxybutynin is often discontinued due to significant side effects. This is hypothesized to be caused by its metabolite. The purpose of the study was to determine whether intravaginal oxybutynin administration leads to fewer anticholinergic side effects than oral oxybutynin. Additionally, the pharmacokinetics (PK), safety, and tolerability were compared.

**Methods:**

The study had a single-blind, placebo-controlled, three-way cross-over design in 24 healthy women. Participants randomly received repeatedly 2.5 mg intravaginal oxybutynin via the MedRing, 5 mg oral oxybutynin, and placebo. Anticholinergic side effects were assessed with the NeuroCart test battery. Additionally, quantitative electro-encephalography (qEEG), salivary flow, dry mouth symptoms, pharmacokinetics, safety, and tolerability were assessed.

**Key findings and limitations:**

Neither intravaginal nor oral oxybutynin demonstrated a significant effect on the adaptive tracking test compared to placebo. However, both oxybutynin administration routes resulted in broad qEEG amplitude decreases. Participants reported less dry mouth symptoms, and the saliva weight was significantly higher after intravaginal oxybutynin (estimated difference, 0.51 g [95% confidence interval, 0.16–0.87], *p* = 0.006). Intravaginal oxybutynin led to a ∼10-fold lower metabolite/parent ratio and was generally safe and well-tolerated.

Limitations include the lack of measured cognitive effect in this population, and the ultimately single-blind study conduct because of subtle differences in the appearance of the ring.

**Conclusions and clinical implications:**

This study provides a solid basis for intravaginal oxybutynin via the MedRing as an alternative route of administration. Intravaginal oxybutynin could be considered an alternative to reduce side effect–related discontinuation rates.

## Take Home Message

There was no difference in cognitive side effects between the three treatments. Intravaginal administration of oxybutynin led to favourable metabolite/parent ratios and less sensation of a dry mouth. Intravaginal oxybutynin could be considered an alternative to reduce side effect-related discontinuation.

ADVANCING PRACTICE **What does this study add?**

Intravaginal administration of oxybutynin results in a lower frequency of dry mouth sensation, and a higher saliva weight compared to oral administration. This aligns with the ∼10-fold lower metabolite/parent ratio observed after intravaginal administration. Moreover, no significant local adverse effects were observed, in contrast to those reported with transdermal formulations. Therefore, intravaginal oxybutynin administration could be considered an alternative route of administration due to the favorable pharmacodynamic and pharmacokinetics effects, that may reduce side effect–related discontinuation.

**Clinical Relevance**

Oxybutynin remains an effective treatment for overactive bladder, but its use is limited by anticholinergic adverse effects, particularly dry mouth, which frequently contributes to treatment discontinuation. By bypassing first-pass hepatic metabolism and reducing exposure to N-desethyloxybutynin, intravaginal oxybutynin may improve tolerability while maintaining systemic drug exposure. The absence of relevant local adverse effects in this early study is encouraging and suggests a potential advantage over transdermal formulations. However, these results were obtained in healthy young women, and further studies in patients with overactive bladder, including older and more vulnerable populations, are needed before clinical implementation. Associate Editor: Professeur Véronique Phé.

**Patient Summary**

In this study we compared the administration of oral and intravaginal oxybutynin in healthy women. We found that certain side effects, like a dry mouth, were less present after intravaginal administration. Compared to oral administration, we conclude that intravaginal administration of oxybutynin could be considered a favorable administration route.

## 1 Introduction

While oxybutynin is the most efficacious oral antimuscarinic treatment to reduce incontinence episodes in patients with an overactive bladder (OAB), oxybutynin is often discontinued due to significant side effects [1]. Oxybutynin is predominantly prescribed as an oral slow- or immediate release tablet, dermal patch, intravesical instillation, or topical gel [2]. It is hepatically metabolized into the primary metabolite N-desethyloxybutynin (DEOB), which results in an oral bioavailability of approximately 6% [2], [3]. By circumvention of the hepatic first-pass effect, bioavailability can be increased to 80% in transdermal administration and results in lower concentrations of DEOB [4]. DEOB is a biologically active metabolite, and its concentration is hypothesized to be related to anticholinergic side effects of oxybutynin [5]. Such side effects are experienced in more than 80% of the oral oxybutynin users, which results in discontinuation rates between 33% and 71% [6], [7]. Transdermal oxybutynin is discontinued in up to 25% of the users, because of local side effects, such as itching, erythema, and skin rash [4], [5], [6], [8]. Consequently, to reduce side effect–related discontinuation rates of oxybutynin, an alternative route of administration has gained interest.

The vaginal mucosa has a highly permeable epithelium and a dense vascular network to enable rapid absorption of small molecules such as oxybutynin, which characterizes an alternative administration route [9], [10], [11], [12], [13], [14], [15], [16]. In a first-in-human study, De Laat et al reported adequate intravaginal absorption of a single dose of 3 mg oxybutynin via the MedRing [17]. This medical device is developed for intravaginal drug delivery. Intravaginal administration of oxybutynin circumvents both hepatic first-pass metabolism and transdermal administration limitations [11], [12], [14], [15], [16], [18], [19], [20].

According to Balk et al [21], a dry mouth is the most frequently reported and most burdensome anticholinergic side effect of oxybutynin, by occupation of the muscarinic-3-receptors (M3R) in the parotid gland [5]. DEOB occupies the M3R to a greater extent and for a longer period, which results in DEOB concentration–related dry mouth side effects [5]. In transdermal oxybutynin, lower concentrations of DEOB result in less dry mouth side effects [22]. Hence, intravaginal oxybutynin potentially reduces dry mouth side effects.

Oxybutynin-related cognitive side effects have extensively been described in elderly patients or those with a high risk of cognitive impairment [21], [23], [24], [25], [26], [27], [28]. The pharmacodynamic (PD) mechanism that underlies these side effects is supported by decreased amplitudes in quantitative electro-encephalography (qEEG) caused by oxybutynin, DEOB, or both [29], [30]. However, cognitive side effects of intravaginal administration have not been quantified.

The purpose of the study was to determine whether intravaginal oxybutynin administration leads to fewer anticholinergic side effects than oral oxybutynin. Furthermore, the pharmacokinetics (PK), safety, and tolerability of both administrations were compared.

## 2 Materials and methods

### 2.1 Participants

The required sample was 24 female participants as justified in the ‘*sample size calculation and statistical analysis section’* below. Before study-specific activities, all participants were asked for written informed consent. If participants were aged 18–45 yr and in general good health, they were included. Participants were excluded if they were a virgin, had intra- or transvaginal surgery that could interfere with MedRing placement or oxybutynin absorption, or had vaginal penetration or a withdrawal bleeding 24 h before oxybutynin administration.

The study was approved by the Medical Ethical Committee Leiden-Delft-Den Haag and was conducted according to the International Conference of Harmonization and Good Clinical Practice and the Declaration of Helsinki. The trial was registered in the EudraCT (2022-001986-12) and the Dutch Trials Registry (NL81624.058.22).

### 2.2 Clinical study design

This study was designed as a prospective, double blind, placebo-controlled, three-way cross-over study to compare intravaginal versus oral oxybutynin administration. Oxybutynin was administered either intravaginally via the MedRing Alpha 2.0 developed by LiGalli B.V. (Leiden, The Netherlands), hereafter referred to as the MedRing, or orally as an encapsulated oxybutynin tablet. An empty ring similar in appearance to the MedRing was used as an intravaginal placebo ring. Placebo capsules were used as an oral placebo.

**Fig. 1:**
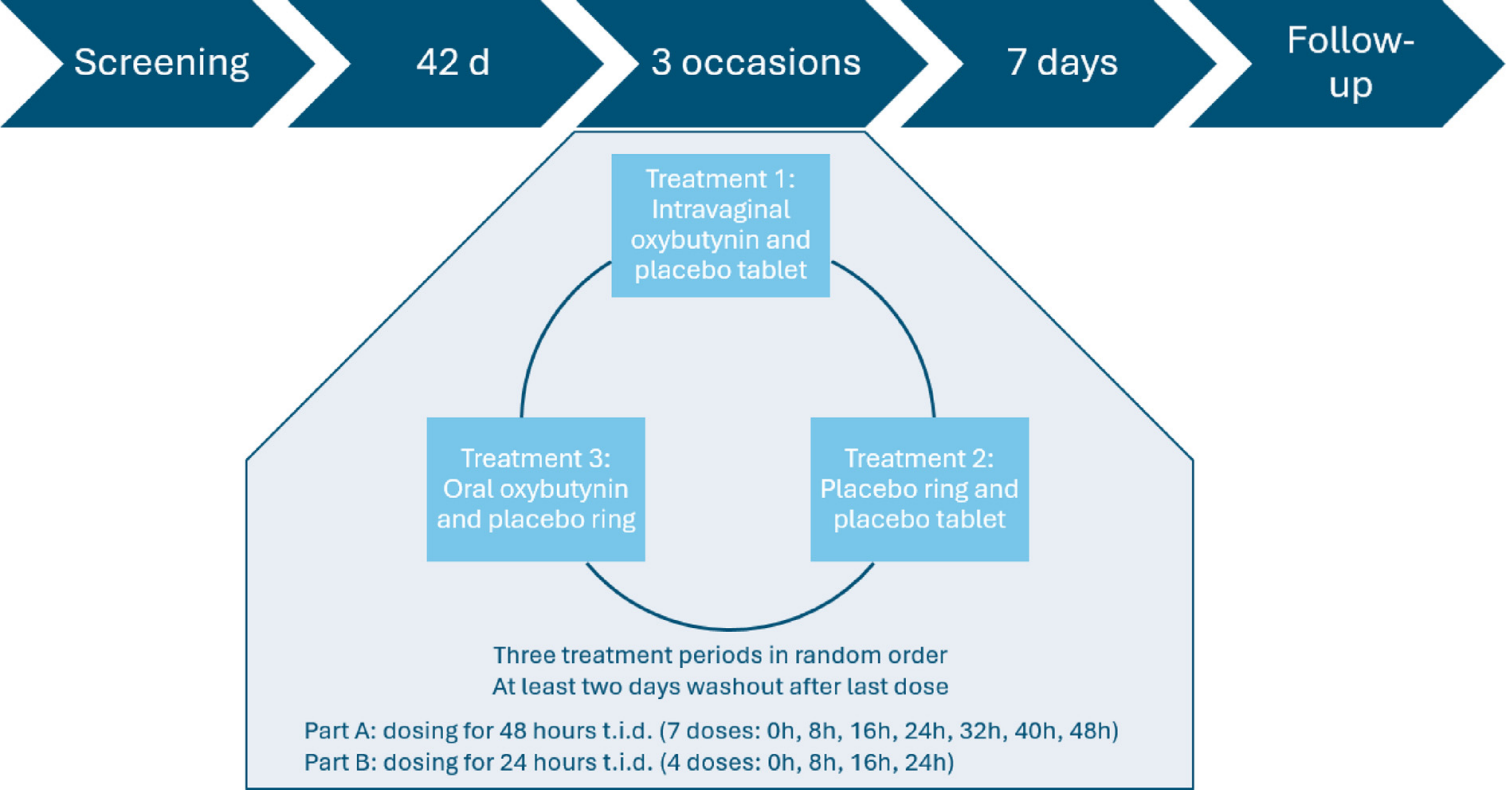
The study design. Each participant received (1) intravaginal oxybutynin and placebo tablet, (2) placebo ring and placebo tablet and (3) oral oxybutynin and placebo ring in randomized order. Part A of the study consisted of 8 participants, who received 7 doses in 48 hours and part B consisted of 16 participants, who received 4 doses in 24 hours. t.i.d. = three times a day; h = hours.

Participants were screened for eligibility prior to dosing. The study consisted of three clinical periods, each separated by a washout period of at least 2 d (Fig. 1). During each period, participants received a different treatment, administered three times a day (t.i.d.). Part A consisted of seven doses in 48 h in eight participants and part B consisted of four doses in 24 h in 16 participants. Part A of the study was conducted first, and part B was performed after a planned interim analysis to confirm the expected PK-profile. The randomly assigned treatments were (1) a MedRing and placebo capsules, (2) a placebo ring and oxybutynin capsules, or (3) a placebo ring and placebo capsules. The randomization was generated by a study-independent Centre for Human Drug Research (CHDR) statistician in SAS (version 9.4), while the investigator, study staff, participants, and sponsor were blinded. Unblinded study personnel calibrated the MedRings before insertion. These unblinded study personnel were not involved in any other study-activity. Seven days after the last clinical visit, participants had a follow-up visit.

The MedRings used in this study are medical devices for intravaginal oxybutynin administration (Supplementary Fig. 1) [17]. The MedRings were pre-filled with 100 mg/ml solution of oxybutynin hydrochloride by Ardena (Ghent, Belgium). The MedRing administers the required 2.5 mg dose in a bolus directly onto the vaginal mucosa. The MedRings were programmed wirelessly via a Bluetooth signal for drug delivery from an external device. Prior to insertion, the MedRings were primed. Dosing accuracy was assessed with a calibration test both prior to insertion and after retrieval.

### 2.3 Methods

During each period, multiple measurements were performed to assess the short-term PD effects using the standardized and validated NeuroCart test battery, hereafter referred to as NeuroCart, for central nervous system effects and peripheral anticholinergic effects [31]. The NeuroCart consisted of eight computerized tests for anticholinergic side effects: the adaptive tracking for sustained attention and hand-eye coordination, N-back test for working memory, saccadic and smooth pursuit eye movements for the level of sedation, pupillometry for autonomic nervous system functioning, body sway for postural stability, visual analog scales for changes in subjective states, visual verbal learning test for learning behavior. Additionally, qEEG was performed with eyes open and eyes closed to assess the effect on the alpha, beta, delta, gamma, and theta power waves. Saliva flow, a dry mouth questionnaire, pulse rate, and visual near point acuity tests were performed for other anticholinergic side effects (Supplementary Methods). At baseline, the NeuroCart measurements were assessed twice and the other PD measurements once. During each period, all PD measurements were repeated multiple times up to 30 h postdose.

During the clinical period, multiple oxybutynin and DEOB PK samples were drawn. These measurements were quantified in plasma with a lower limit of quantification of 0.100 ng/ml using a validatedliquid chromatography tandem mass spectrometry (LC-MS/MS) method by Ardena Bioanalytical Laboratories (Assen, The Netherlands).

Tolerability and safety were monitored via treatment-emergent adverse events (TEAEs), vital signs, laboratory tests (hematology, clinical chemistry, and urinalysis), an electrocardiogram, a tolerability questionnaire, and a physical and gynecological examination.

### 2.4 Dose selection

Data of the previous study with the MedRing and the registered oral oxybutynin dose regime of 5 mg t.i.d. were used to determine the dose in a population PK (popPK) model [17]. Clinical trial simulations were performed to determine a dose that reached transdermal target concentrations [5]. An intravaginal dose of 2.5 mg oxybutynin t.i.d. was estimated to be in the area under the curve of the dosing interval (AUC_tau_) target range of 25–35 ng*h/ml as calculated from the efficacious transdermal oxybutynin dose [3], [17], [22], [32]. Additionally, a dose of 2.5 mg was estimated to be slightly above the C_max_ and AUC of oral oxybutynin. Moreover, based on these popPK model simulations, oral oxybutynin or placebo would be administered 1 h after intravaginal oxybutynin or placebo to align the PD measurements during the upward slope near the T_max_ of both administration routes.

### 2.5 Sample size calculation and statistical analysis

In the sample size calculation, the single comparison of interest was intravaginal oxybutynin versus oral oxybutynin. The placebo comparison was not included in the sample size determination. For the adaptive tracking test, a sample size of 24 will have 80% power to detect a difference in means of 2 (%) using a paired t-test with a 0.050 two-sided significance level. This percentage was based on a sample size calculation with an estimated intra subject variance (standard deviation of 3.5 [%]) of earlier studies with adaptive tracking in healthy female volunteers. As only a noteworthy difference in anticholinergic side effects will be clinically relevant, a smaller sample size due to non-evaluable participants or dropouts, will be sufficient to answer the primary objective. The same applies for a higher standard deviation than 3.5 (%).

The statistical analysis for PD, PK, safety, and tolerability outcomes is outlined in the Supplementary Methods.

## 3 Results

### 3.1 Study population and baseline

The study was conducted between March 31, 2023, and August 3, 2023, at CHDR, Leiden, The Netherlands. After providing informed consent, 57 healthy women were screened and 24 participants were randomized, enrolled, and divided over part A and B. All included participants completed the study. The demographics and baseline characteristics of the participants are outlined in Table 1.

**Table 1:** Demographics

Characteristic	All participants (part A and B)
Age (yr)	24 (21–26)
Height (cm)	171 (167–175)
Weight (kg)	66 (60–74)
BMI (kg/m^2^)	22 (20–25)
Race, *n* (%)	
• White	20 (83)
• Black or African American	1 (4)
• Mixed	3 (13)

n = number of participants.

Estimates were given as median (quartile 1, quartile 3) or frequencies (percentage).

Two physicians, who inserted the MedRings, were able to distinguish the MedRings filled with oxybutynin from the placebo rings used in the placebo and oral oxybutynin arm, because the inlet of the filled MedRings was closed with glue. While the study was double blind in design, these two physicians were unblinded during the study for one of the three treatment allocations. However, these physicians were merely involved in the safety and tolerability assessments. The participants and all other study personnel remained blinded. Nevertheless, ultimately, the study is considered single-blind.

### 3.2 Interim analysis

The results of the interim analysis confirmed that the concentrations of oxybutynin and DEOB were as expected in the popPK model. These results justified to continue with part B as planned.

### 3.3 NeuroCart test battery

**Fig. 2:**
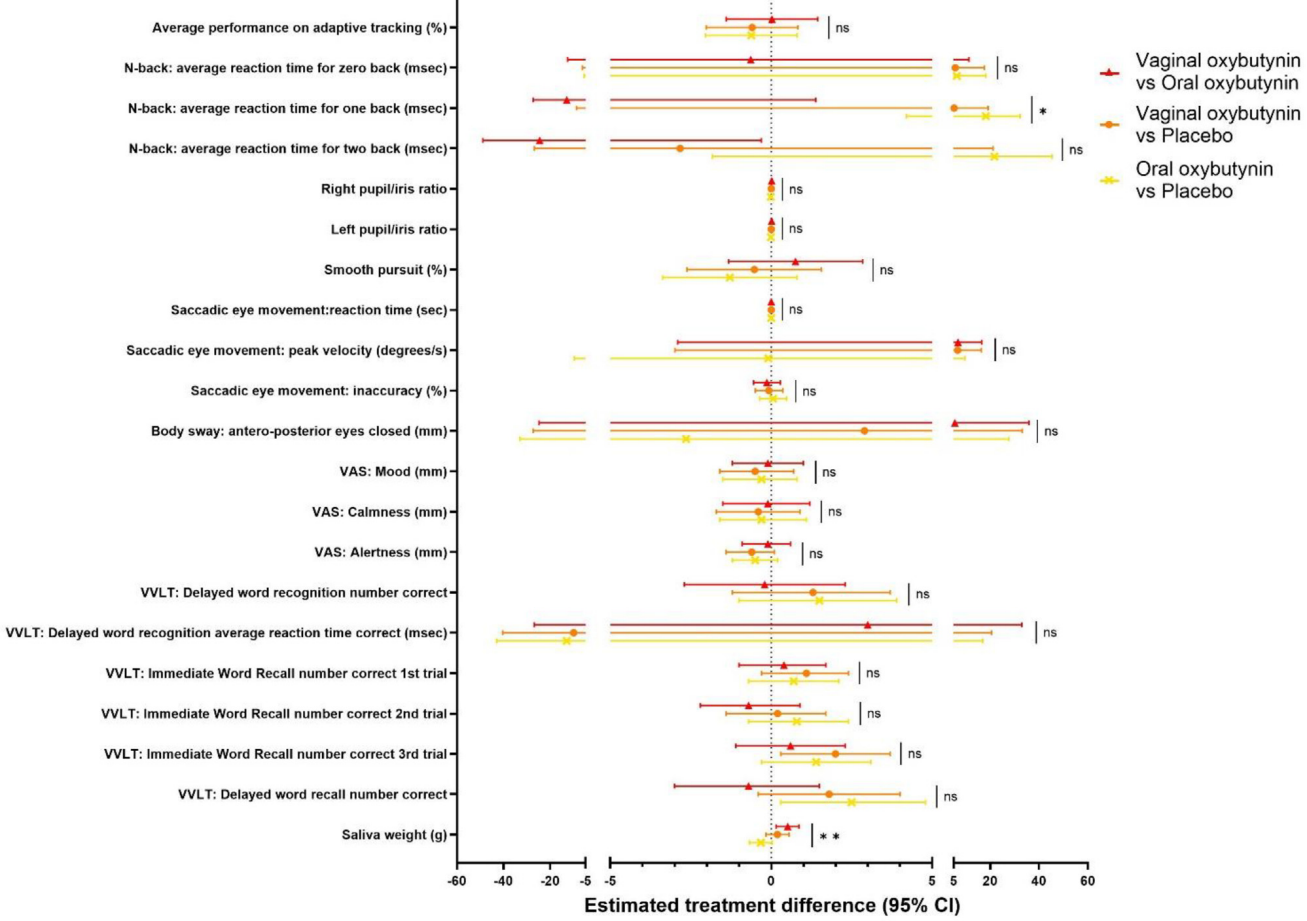

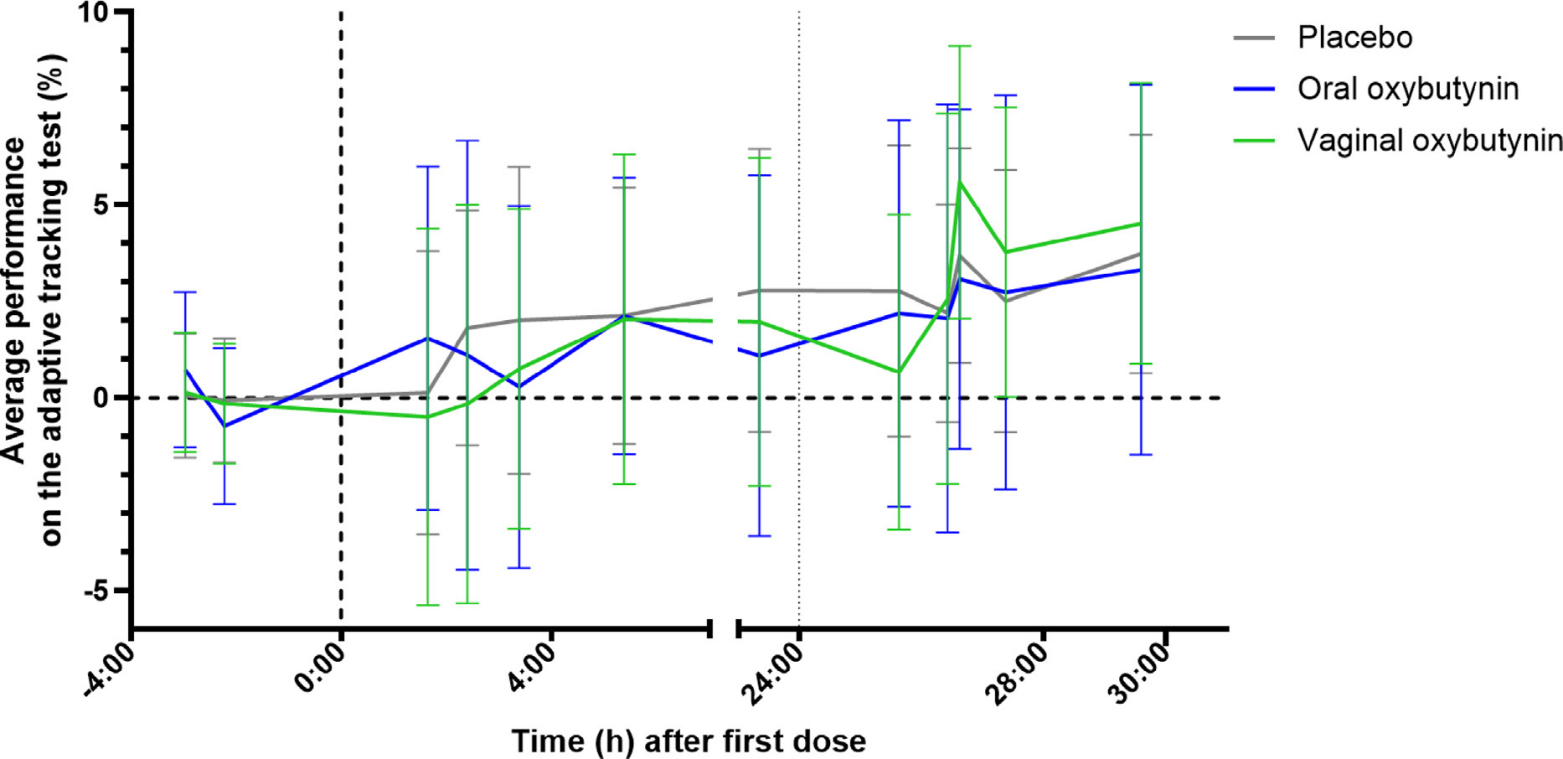

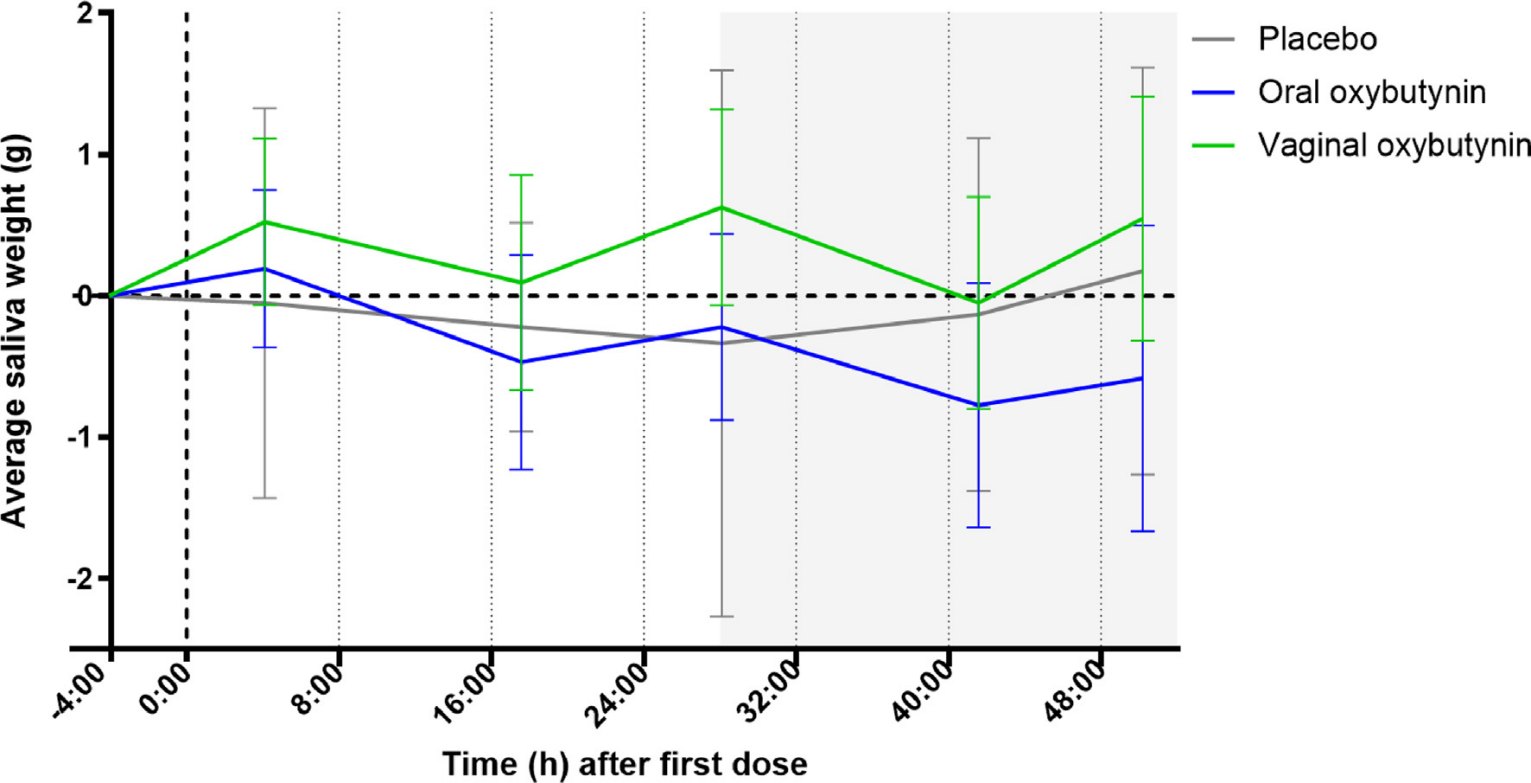

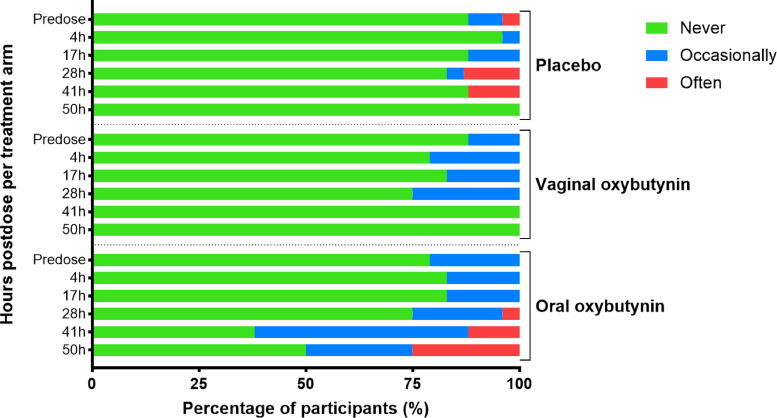
Pharmacodynamic results of intravaginal oxybutynin, oral oxybutynin and placebo Description: Results of estimated treatment differences for each NeuroCart test and saliva weight (a). There was no significant difference in the LSMeans of the adaptive tracking test (b). A significant difference was observed in the 3-way comparison of the average reaction time in the N-back test (one back) and the LSMeans of the saliva weight (c). There was no correction performed for multiple testing in the linear mixed-effects ANCOVA model. In the tolerability questionnaire, more participants reported a dry mouth after oral oxybutynin (d). (A) Estimated treatment differences (95% CIs) for each NeuroCart test and saliva weight from a linear mixed-effects ANCOVA model, including 2-way and 3-way comparisons. CIs = confidence intervals; msec = milliseconds; sec = seconds; mm = millimeters; VAS = visual analogue scale; VVLT = visual verbal learning test; g = gram; ns = not significant. Symbols: * overall p-value of 3-way comparison *p* = 0.037; ** overall *p*-value of 3-way comparison *p* = 0.019. Statistical analysis: estimated treatment differences (95% CIs) and *p*-values were calculated using a linear mixed effects ANCOVA model of covariance and corrected for (double) baseline measurements. (B) LSMeans of performance on the adaptive tracking (%) estimated differences (95% CIs), correction for double baseline (CFB) from a linear mixed-effects ANCOVA model. CFB = correction for double baseline; CIs = confidence intervals; h = hours; LSMeans = Least Squares Means. X-axis: Striped line = first intravaginal dose; dotted line = consecutive intravaginal dose. Statistical analysis: LSMeans, estimated differences (95% CIs) and p-values were calculated using a linear mixed effects ANCOVA model of covariance and corrected for double baseline measurements. Vaginal oxybutynin versus oral oxybutynin had an ED of 0.026% (95% CI, −1.4 to 1.4), *p* > 0.9. Oral doses were administered 1 hour past intravaginal doses. (C) LSMeans of the saliva weight (grams) estimated differences (95% CIs), correction for baseline (CFB) from a linear mixed-effects ANCOVA model. g = grams; CFB = correction for baseline; CIs = confidence intervals; h = hours; LSMeans = Least Squares Means. X-axis: Striped line = first intravaginal dose; dotted line = consecutive intravaginal doses; grey shaded background = only participants in part A, in total 8 participants. Statistical analysis: LSMeans, estimated differences (95% CIs) and p-values were calculated using a linear mixed effects ANCOVA model of covariance and corrected for baseline measurements. Vaginal oxybutynin versus oral oxybutynin had an ED of 0.51 g (95% CI, 0.16–0.87), *p* = 0.006. Oral dosing was 1 hour after intravaginal dosing. (D) Answers to the question “my mouth feels dry”. h = hours. 41h and 50h represent only participants in part A, in total 8 participants.

In Fig. 2, the results of the NeuroCart tests are outlined. There were no significant differences between the three treatments for most tests (Fig. 2a). Although intravaginal and oral oxybutynin showed no significant difference in the adaptive tracking test, they also did not differ from placebo (Fig. 2b). In the one-back test there was a significant difference between the three treatments, but not between pairs (Fig. 2a). The average reaction time in the two-back test was significantly shorter after intravaginal compared to oral administration (estimated difference (ED) 25 msec (95% confidence interval [CI], −49 to −0.3), *p* = 0.047).

### 3.4 Other pharmacodynamic outcomes

Although both intravaginal and oral oxybutynin had decreased amplitudes of various frequency bands in alpha, delta and theta power waves compared to placebo, there were no significant differences between intravaginal and oral oxybutynin.

While saliva weight did not differ between intravaginal oxybutynin and placebo, after oral oxybutynin administration compared to intravaginal, it was significantly reduced (ED, 0.51 g (95% CI, 0.16–0.87), *p* = 0.006; Fig. 2c). Moreover, after intravaginal oxybutynin, a dry mouth was reported less frequently, with a lower severity and a shorter duration (Fig. 2d).

There were no significant differences between the three treatment arms in the visual acuity test or pulse rates.

### 3.5 Pharmacokinetics

**Fig. 3:**
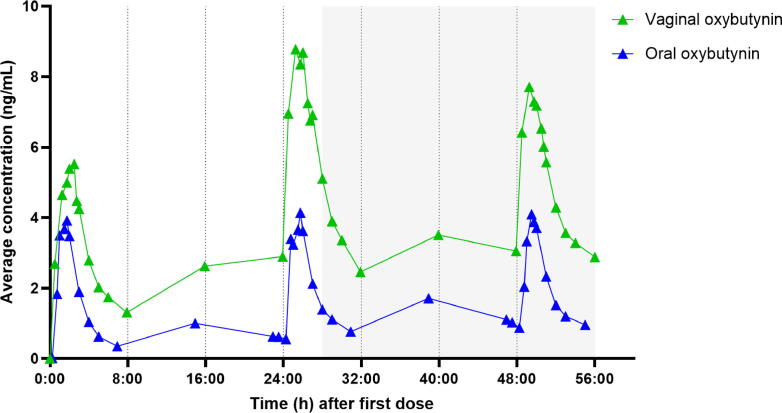

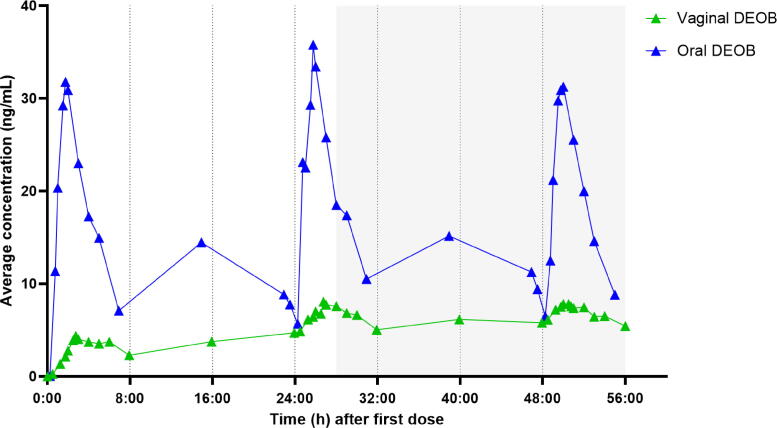

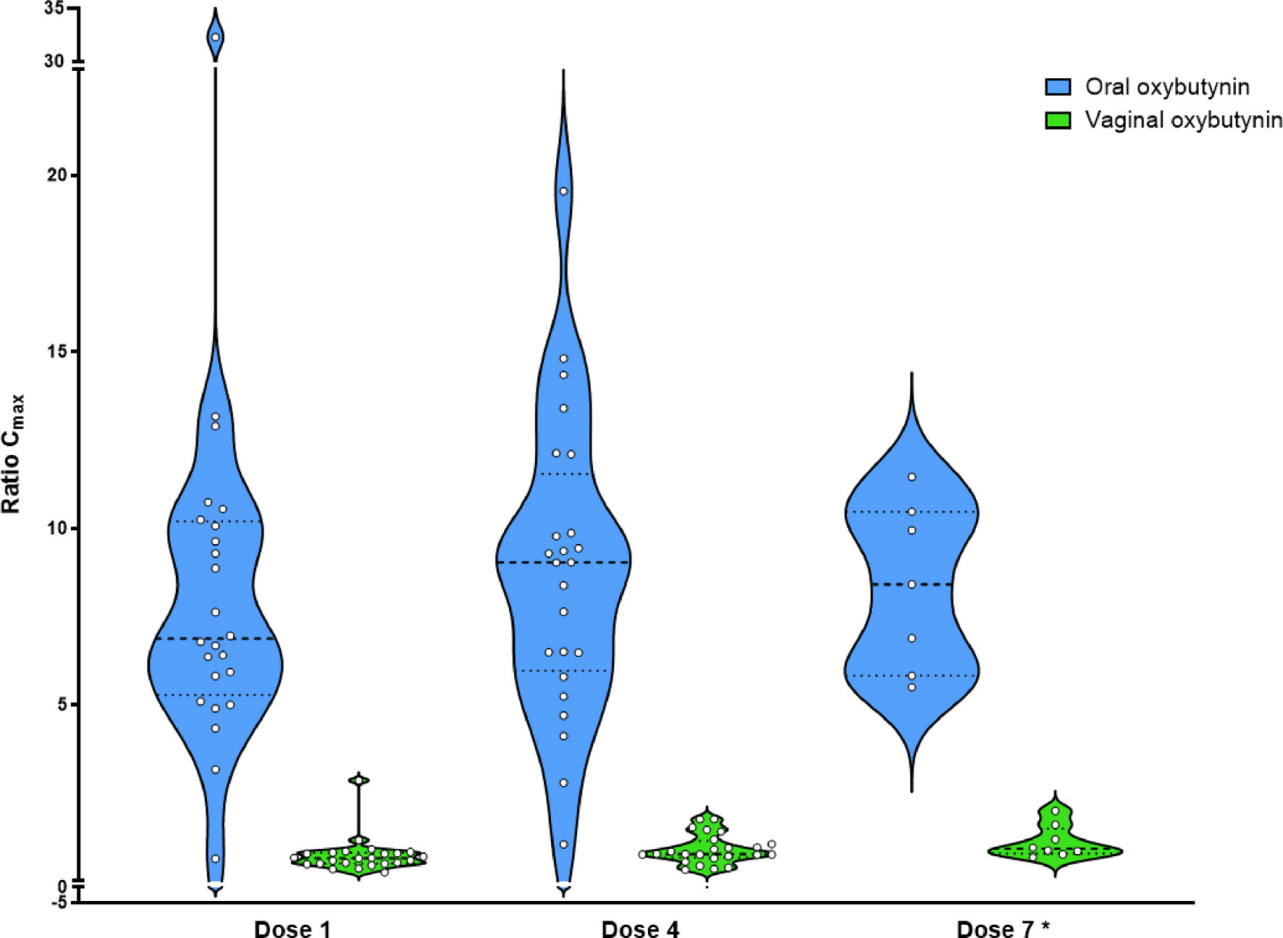
Pharmacokinetic results of oxybutynin and DEOB per treatment Description: Intravaginal oxybutynin administration resulted in higher average oxybutynin plasma concentrations (a) and lower DEOB plasma concentrations (b). This resulted in a ∼10 fold lower metabolite/parent Cmax ratio (c). (A) Average concentrations of oxybutynin in plasma (ng/ml) after intravaginal and oral oxybutynin administration. X-axis: dotted line = consecutive doses; grey-shaded background = only participants in part A, in total 8 participants. ng/mL = nanograms per milliliter; h = hour. (B) Average concentrations of N-desethyloxybutynin (DEOB) in plasma (ng/ml) after intravaginal and oral oxybutynin administration. X-axis: dotted line = consecutive doses; grey-shaded background = only participants in part A, in total 8 participants. DEOB = N-desethyloxybutynin; ng/ml = nanograms per milliliter; h = hour. (C) Ratio Cmax metabolite/parent per dose per treatment. Cmax = maximum concentration. Symbols: striped line = median; dotted line = quartiles; white dots = individual values; * = only participants in part A, in total 8 participants.

After four doses, the median peak concentrations of intravaginal oxybutynin occurred at a T_max_ of 2.0 h and for DEOB at 2.9 h and for oral oxybutynin the T_max_ was at 1.0 h and for DEOB at 1.5 h. Compared to oral administration, the C_max_ and AUC_0-7h_ of oxybutynin were higher after intravaginal administration, while these were for DEOB lower (Table 2a-b and Fig. 3a-b). This resulted in a ∼10-fold lower metabolite/parent C_max_ ratio after intravaginal administration (Fig. 3c).

**Table 2:** Pharmacokinetics results of oxybutynin and DEOB per treatment

		Intravaginal 2.5 mg t.i.d.				Oral 5 mg t.i.d.							
		Oxybutynin		DEOB		Oxybutynin					DEOB		
Parameter	Dose	*N*	Mean	SD	*N*	Mean	SD	*N*	Mean	SD	*N*	Mean	SD
C_max_ (ng/ml)	1	24	5.5	3.2	24	4.2	5.6	24	5.4	2.7	24	37	13
	4	24	9.9	4.3	24	8.3	5.4	24	5.3	3.1	24	39	16
	7	8	7.9	3.7	8	8.2	3.5	7	4.7	1.6	7	36	7.1

AUC_0–4h_ (ng * h/ml)	1	24	15	8.2	24	9.3	9.8	23	8.6	3.5	23	81	32
	4	22	29	10	22	25	11	15	11	5.1	22	93	41
	7	8	27	10	8	29	12	7	10	3.9	7	90	29

AUC_0–7h_ (ng * h/ml)	1	23	21	11	23	19	18	23	10	4.0	23	113	43
	4	7	33	7.3	7	46	17	6	14	5.2	7	148	62
	7	8	35	12	8	48	21	7	14	5.2	7	130	45

t.i.d. = three times a day; DEOB = N-desethyloxybutynin; N = number of participants; SD = standard deviation; C_max_ = maximum concentration; ng = nanograms; mL = milliliters; AUC = area under the curve.

### 3.6 Safety and tolerability

In total, 103 TEAEs were reported, of which 87 were considered related to the treatment. There were 28 related TEAEs after intravaginal oxybutynin, 38 related TEAEs after oral oxybutynin and 21 related TEAEs after placebo (Supplementary Table 1). The most common TEAEs after intravaginal oxybutynin included fatigue, abdominal pain, and headache. Of the six abdominal pain TEAEs, three lasted <1 hr, two lasted 6 hr and one started 3 d after the first dose. One participant reported increased discharge and vulvovaginal pruritus after intravaginal oxybutynin. All TEAEs were considered mild, transient, and resolved without sequelae.

Based on the questionnaires, inserting the MedRing was not uncomfortable, and its presence was unnoticeable. Removal of the MedRing or placebo ring was uncomfortable in 13 participants, but not painful. Moreover, all calibration output tests were successful.

## 4 Discussion

The purpose of the study was to determine whether intravaginal oxybutynin administration leads to fewer anticholinergic side effects than oral oxybutynin. Furthermore, the PK, safety and tolerability of both administrations were compared. Although the primary outcome was not met the present study suggested favorable PK with potentially a beneficial side effect profile after intravaginal oxybutynin administration compared to oral oxybutynin.

In the present study, intravaginal oxybutynin resulted in a significant difference in a dry mouth compared to oral oxybutynin, in favor of intravaginal oxybutynin. The advantage for intravaginal oxybutynin compared to oral oxybutynin was seen for both saliva weight and the frequency, severity, and duration of a dry mouth sensation. These findings contrast with those of Balk et al, as the present study indicates that after intravaginal oxybutynin, a dry mouth was not the most commonly observed side effect [21]. Despite a lower absolute dose, higher oxybutynin concentrations were measured after intravaginal administrations. Moreover, by bypassing the first-pass effect, the C_max_ metabolite/parent ratio was reduced by ∼10-fold. As hypothesized, this favorable ratio, due to lower DEOB concentrations, resulted in a decrease in the sensation of a dry mouth.

Although differences in qEEG data were observed between placebo and both oxybutynin treatments, no differences in cognitive side effects were detected across the three treatments. This absence of effect was unexpected, as the calculated difference in T_max_ from the popPK model is consistent with our findings and short-term cognitive side effects have previously been reported after oral oxybutynin administration [6]. Our findings contrast with effects that are reported for the muscarinic antagonist scopolamine in the adaptive tracking test [31]. The lack of effect for oxybutynin in this functional test could be a result of differences in muscarinic receptor potency and affinity [31]. Potentially, with higher dosages of oxybutynin, an effect could have been observed. However, as the current intravaginal dose reflects the used clinical transdermal dose, a higher dose would not have been relevant for clinical practice.

Another possible explanation is that female adolescents and young adults are less sensitive to oxybutynin-related cognitive side effects, although extensively described in other populations in previous research [21], [23], [24], [25]. While anticholinergics-related cognitive side effects are reported in children, these side effects were limited to cases of oxybutynin overdose [33]. Had our population been older, it is possible that the adaptive tracking test would have shown a significant difference. Our findings suggest that cognitive side effects after therapeutic doses of oxybutynin do not occur in young, healthy women, irrespective of the administration route.

After intravaginal oxybutynin, participants seemed to have a better working memory compared to oral oxybutynin in one out of three tests. No differences were seen in the other two working memory tests. Additionally, a previous study in the elderly reported no effect of twice-daily oral oxybutynin 5 mg in the first 6 hr postdose on working memory [28]. Therefore, these results should be interpreted carefully.

Our findings may suggest that intravaginal oxybutynin may be preferred over transdermal oxybutynin administration, because our study did not show significant local side effects, in contrast to the findings reported for transdermal oxybutynin administration by Baldwin et al [5]. Despite this difference, our findings align with those of Baldwin et al regarding its advantageous PK plasma levels [5]. Based on their data of a total daily dose of 3.9 mg, the mean metabolite/parent C_max_ ratio was 1.5, which is comparable to the observed metabolite/parent C_max_ ratio of 0.9 after intravaginal administration.

In general, the MedRing was well-tolerated. Six of 24 participants experienced abdominal pain, which was transient and mild in severity. Slightly over half of the women experienced discomfort during removal, though none reported pain. It is unclear whether this discomfort was due to the MedRing or the vaginal examination itself, as such discomfort is not uncommon in clinical practice [34]. Furthermore, this favorable local tolerability might contribute to a higher treatment adherence compared to transdermal administration due to the lack of application site reactions.

Clearly, there are some limitations to this study. Firstly, although designed as double blind study, the study was ultimately conducted single-blind because the MedRing was not identical to the placebo ring. Therefore, the interpretation of safety and tolerability assessments could be biased. However, since the unblinded physicians were not involved in the collection of PD or PK data, the double blind design was maintained for these objectives. Secondly, this study included relatively young, healthy women, thus the interpretation for OAB-patients should be done cautiously. Especially since cognitive side effects following oxybutynin administration have not previously been described in this population, the lack of observed differences in cognitive side effects may therefore not be generalizable to older women.

## 5 Conclusion

This study provides a solid basis for intravaginal oxybutynin as an alternative route for oxybutynin administration. While achieving higher oxybutynin and lower DEOB concentrations, intravaginal oxybutynin results in less dry mouth side effects than oral administration. Moreover, there is a potential dual benefit due to a favorable local tolerability when compared to transdermal administration. Therefore, intravaginal oxybutynin via the MedRing could be considered an alternative to reduce side effect–related discontinuation rates.

  ***Author contributions:*** Naomi B. Klarenbeek had full access to all the data in the study and takes responsibility for the integrity of the data and the accuracy of the data analysis.

  *Study concept and design*: Meijs, Pagan, Klarenbeek, Otto.

*Acquisition of data*: Meijs, Pagan, Klarenbeek, Somohardjo.

*Analysis and interpretation of data*: Peltenburg, Meijs, Klarenbeek, Tibboel, Somohardjo, Strugala, Juachon.

*Drafting of the manuscript*: Peltenburg, Meijs.

*Critical revision of the manuscript for important intellectual content*: Pagan, van Uhm, Elzevier, Burggraaf, Hellebrekers, Klarenbeek.

*Statistical analysis*: Juachon, Strugala, Otto, Tibboel.

*Obtaining funding*: Klarenbeek.

*Administrative, technical, or material support*: Meijs, Pagan.

*Supervision*: Klarenbeek.

*Other* (specify): None.

  ***Financial disclosures:*** Naomi B. Klarenbeek certifies that all conflicts of interest, including specific financial interests and relationships and affiliations relevant to the subject matter or materials discussed in the manuscript (eg, employment/affiliation, grants or funding, consultancies, honoraria, stock ownership or options, expert testimony, royalties, or patents filed, received, or pending), are the following: None.

  ***Funding/Support and role of the sponsor:*** The study was funded by LiGalli BV. The study was designed as a collaborative effort between CHDR and LiGalli BV. CHDR was responsible for the conduct of the study and monitoring did the TAPAS group (Laren, the Netherlands). Collection, management, analysis, and interpretation of the data was done by CHDR. The preparation of the manuscript was done by SP and AM; all coauthors and the sponsor reviewed and approved the final manuscript. Finally, we would like to thank the volunteers for their participation.

## Appendix A Supplementary data

Supplementary Data 1 The figure of the MedRing, the methods of pharmacodynamic assessments and statistical analysis, and the table of the summary of adverse events.
