# Supplementary material for: Intravaginal Delivery of Oxybutynin: An Alternative Administration Route to Improve its Pharmacokinetic and Pharmacodynamic Effects
Source: Eur Urol Open Sci. 2026 Jul 23;91:12–22. doi: 10.1016/j.euros.2026.07.004 (PMC13425807; doi:10.1016/j.euros.2026.07.004)
Supplement: Supplementary Data 1 — The figure of the MedRing, the methods of pharmacodynamic assessments and statistical analysis, and the table of the summary of adverse events. [file mmc1.docx]

**Supplementary figure 1:** The MedRing Alpha 2.0


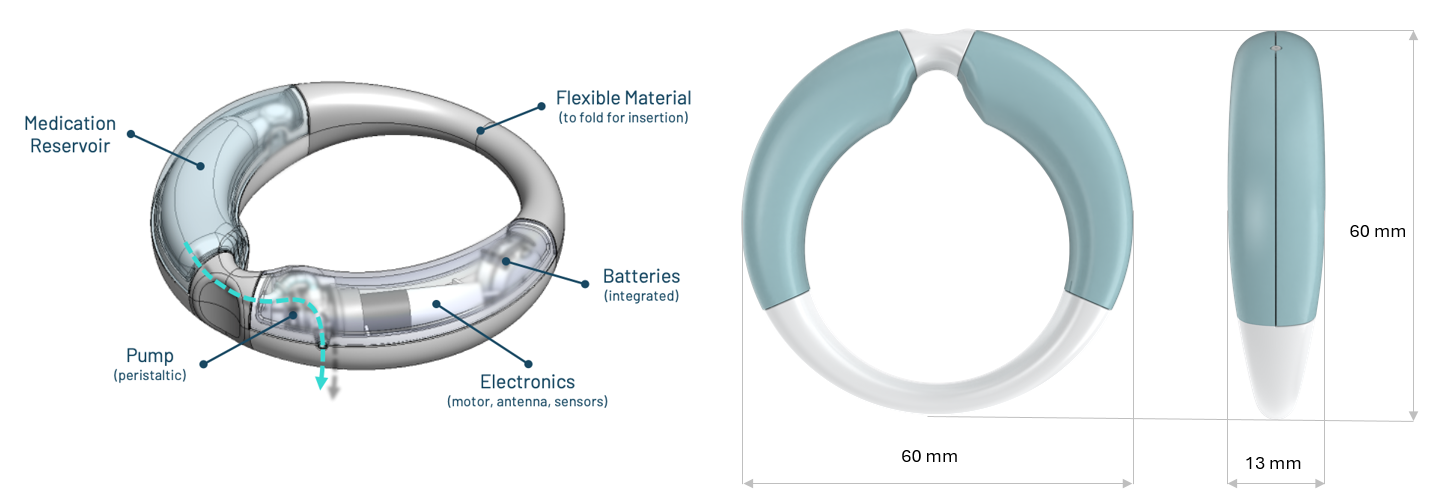


**Supplementary methods**

**PD assessments**

Saliva flow was measured using Sarstedt neutral Salivettes (Sarstedt, Etten Leur, The Netherlands) collection tubes, provided with dental rolls. The saliva production was estimated by measuring the increase in weight of three dental rolls put into the oral cavity over 3 minutes of time.

The visual near point acuity was determined using a Sloane Letter Near Vision Card to measure the visual near point acuity and quality of sight. This card was held at 40 centimetres from the participant. Participants were instructed to read the letters out loud. The visual value from the letter card were registered.

The Summated Xerostomia Inventory Dutch questionnaire was translated for the dry-mouth questionnaire to measure xerostomia symptoms in clinical research: “My mouth feels dry when eating a meal”, “My mouth feels dry”, “I have difficulty in eating dry foods”, “I have difficulty swallowing certain foods” and “My lips feel dry”. All questions could be answered with never, occasionally or often.

**Statistical analysis**

PD variables measured repeatedly over time were analysed using a linear mixed-effects analysis of covariance model. Fixed effects were treatment (intravaginal oxybutynin, oral oxybutynin, placebo), period, time, and treatment-by-time interaction and random effects were subject, subject-by-treatment, and subject-by-time to account for within-subject correlation in the crossover design. The average predose (baseline) value was included as a covariate. PD variables measured once per treatment period were analysed using a linear mixed-effects ANCOVA model with treatment and period as fixed effects and subject as a random effect, including the average value as covariate. A variance components covariance structure was used in the mixed model. Pre-specified contrasts were intravaginal oxybutynin versus oral oxybutynin, intravaginal oxybutynin versus placebo, and oral oxybutynin versus placebo. Although placebo was not a pre-specified powered comparison, these comparisons were presented with p-values, but these results were considered exploratory analyses. All calculations were performed using SAS for windows V9.4 (SAS Institute, Inc., Cary, NC, USA).

Results are presented as Least Squares Means (LSMeans), estimated treatment differences, 95% confidence intervals, and p-values. For log-transformed variables, LSMeans are presented as geometric means, and treatment differences were back-transformed and expressed as percentages with corresponding confidence intervals. LSMeans plots were generated for both absolute values and change from baseline. No adjustment for multiple testing was applied due to the exploratory nature of the study, thus all variables other than the primary outcome will be considered exploratory endpoints.

For the PK analysis, the area under the curve from 0 to 7h (AUC_0-7h_), maximum concentration (C_max_), time to attain C_max_ (T_max_) were calculated with a non-compartmental analysis of plasma concentration over time in R (version 4.0.3).

Safety and tolerability outcomes were listed.

**Supplementary table 1:** Summary of Treatment Emergent Adverse Events

|  | **Vaginal oxybutynin (N=24)** | | **Oral oxybutynin (N=24)** | | **Placebo (N=24)** | |
| --- | --- | --- | --- | --- | --- | --- |
| **System Organ Class/  Preferred Term** | Events  N | participants  N (%) | Events  N | participants  N (%) | Events  N | participants  N (%) |
| ANY EVENTS | 35 | 17 (71) | 42 | 17 (71) | 26 | 13 (54) |
| EYE | 1 | 1 (4.2) | 3 | 3 (13) | 1 | 1 (4.2) |
| Dry eye | - | - | 1 | 1 (4.2) | - | - |
| Vision blurred | 1 | 1 (4.2) | 2 | 2 (8.3) | 1 | 1 (4.2) |
| GASTROINTESTINAL | 8 | 8 (33) | 10 | 8 (33) | 4 | 4 (17) |
| Abdominal distension | - | - | 1 | 1 (4.2) | - | - |
| Abdominal pain | 5 | 5 (21) | 1 | 1 (4.2) | 1 | 1 (4.2) |
| Abdominal pain lower | 1 | 1 (4.2) | 1 | 1 (4.2) | 1 | 1 (4.2) |
| Diarrhoea | - | - | 1 | 1 (4.2) | - | - |
| Dry mouth | 1 | 1 (4.2) | 3 | 2 (8.3) | 1 | 1 (4.2) |
| Dry throat | - | - | - | - | 1 | 1 (4.2) |
| Lip dry | - | - | 1 | 1 (4.2) | - | - |
| Nausea | 1 | 1 (4.2) | 2 | 2 (8.3) | - | - |
| GENERAL DISORDERS | 11 | 7 (29) | 9 | 7 (29) | 5 | 4 (17) |
| Catheter site pain | 2 | 2 (8.3) | 1 | 1 (4.2) | - | - |
| Fatigue | 6 | 5 (21) | 6 | 5 (21) | 4 | 4 (17) |
| Feeling hot | - | - | 1 | 1 (4.2) | - | - |
| Influenza like illness | 1 | 1 (4.2) | - | - | - | - |
| Malaise | 1 | 1 (4.2) | 1 | 1 (4.2) | 1 | 1 (4.2) |
| Thirst | 1 | 1 (4.2) | - | - | - | - |
| INJURY AND PROCEDURAL | 1 | 1 (4.2) | - | - | 1 | 1 (4.2) |
| Catheter site pain | - | - | - | - | 1 | 1 (4.2) |
| Muscle strain | 1 | 1 (4.2) | - | - | - | - |
| INVESTIGATIONS | 1 | 1 (4.2) | - | - | 1 | 1 (4.2) |
| Menstruation normal | 1 | 1 (4.2) | - | - | 1 | 1 (4.2) |
| MUSCULOSKELETAL | - | - | - | - | 1 | 1 (4.2) |
| Back pain | - | - | - | - | 1 | 1 (4.2) |
| NERVOUS SYSTEM | 9 | 8 (33) | 11 | 10 (42) | 7 | 5 (21) |
| Dizziness | 1 | 1 (4.2) | 1 | 1 (4.2) | 1 | 1 (4.2) |
| Headache | 5 | 4 (17) | 5 | 4 (17) | 6 | 5 (21) |
| Paraesthesia | 1 | 1 (4.2) | - | - | - | - |
| Somnolence | 2 | 2 (8.3) | 5 | 5 (21) | - | - |
| PSYCHIATRIC | 2 | 2 (8.3) | 4 | 4 (17) | 1 | 1 (4.2) |
| Depressed mood | - | - | 1 | 1 (4.2) | - | - |
| Disturbance in attention | - | - | 2 | 2 (8.3) | - | - |
| Somnolence | 2 | 2 (8.3) | 1 | 1 (4.2) | 1 | 1 (4.2) |
| URINARY | - | - | 1 | 1 (4.2) | 1 | 1 (4.2) |
| Lower urinary tract symptoms | - | - | - | - | 1 | 1 (4.2) |
| Urinary retention | - | - | 1 | 1 (4.2) | - | - |
| GYNAECOLOGICAL AND BREAST | 1 | 1 (4.2) | 1 | 1 (4.2) | 1 | 1 (4.2) |
| Dysmenorrhoea | - | - | 1 | 1 (4.2) | - | - |
| Vaginal discharge | 1 | 1 (4.2) | - | - | - | - |
| Vaginal haemorrhage | - | - | - | - | 1 | 1 (4.2) |
| RESPIRATORY AND THORACIC | - | - | 3 | 2 (8.3) | 2 | 2 (8.3) |
| Dry throat | - | - | 1 | 1 (4.2) | - | - |
| Dysphonia | - | - | 1 | 1 (4.2) | - | - |
| Hiccups | - | - | - | - | 1 | 1 (4.2) |
| Oropharyngeal pain | - | - | 1 | 1 (4.2) | - | - |
| Upper respiratory tract infection | - | - | - | - | 1 | 1 (4.2) |
| SKIN | 1 | 1 (4.2) | - | - | 1 | 1 (4.2) |
| Erythema | - | - | - | - | 1 | 1 (4.2) |
| Vulvovaginal pruritus | 1 | 1 (4.2) | - | - | - | - |
